# Evolution of the osteoblast: skeletogenesis in gar and zebrafish

**DOI:** 10.1186/1471-2148-12-27

**Published:** 2012-03-05

**Authors:** B Frank Eames, Angel Amores, Yi-Lin Yan, John H Postlethwait

**Affiliations:** 1 Institute of Neuroscience, University of Oregon, Eugene, OR 97403-1254, USA; 2 Department Anatomy and Cell Biology, University of Saskatchewan, 107 Wiggins Road, Saskatoon, SK S7N 5E5, Canada

## Abstract

**Background:**

Although the vertebrate skeleton arose in the sea 500 million years ago, our understanding of the molecular fingerprints of chondrocytes and osteoblasts may be biased because it is informed mainly by research on land animals. In fact, the molecular fingerprint of teleost osteoblasts differs in key ways from that of tetrapods, but we do not know the origin of these novel gene functions. They either arose as neofunctionalization events after the teleost genome duplication (TGD), or they represent preserved ancestral functions that pre-date the TGD. Here, we provide evolutionary perspective to the molecular fingerprints of skeletal cells and assess the role of genome duplication in generating novel gene functions. We compared the molecular fingerprints of skeletogenic cells in two ray-finned fish: zebrafish (*Danio rerio*)--a teleost--and the spotted gar (*Lepisosteus oculatus*)--a "living fossil" representative of a lineage that diverged from the teleost lineage prior to the TGD (i.e., the teleost sister group). We analyzed developing embryos for expression of the structural collagen genes *col1a2, col2a1, col10a1*, and *col11a2 *in well-formed cartilage and bone, and studied expression of skeletal regulators, including the transcription factor genes *sox9 *and *runx2*, during mesenchymal condensation.

**Results:**

Results provided no evidence for the evolution of novel functions among gene duplicates in zebrafish compared to the gar outgroup, but our findings shed light on the evolution of the osteoblast. Zebrafish and gar chondrocytes both expressed *col10a1 *as they matured, but both species' osteoblasts also expressed *col10a1*, which tetrapod osteoblasts do not express. This novel finding, along with *sox9 *and *col2a1 *expression in developing osteoblasts of both zebrafish and gar, demonstrates that osteoblasts of both a teleost and a basally diverging ray-fin fish express components of the supposed chondrocyte molecular fingerprint.

**Conclusions:**

Our surprising finding that the "chondrogenic" transcription factor *sox9 *is expressed in developing osteoblasts of both zebrafish and gar can help explain the expression of chondrocyte genes in osteoblasts of ray-finned fish. More broadly, our data suggest that the molecular fingerprint of the osteoblast, which largely is constrained among land animals, was not fixed during early vertebrate evolution.

## Background

Skeletal tissues provide invaluable traits to document vertebrate evolution and to reveal the mechanistic basis for evolutionary change. Two main processes underlie skeletal development: histogenesis--histological differentiation of skeletal tissues--and morphogenesis--acquisition of skeletal element location, shape, and size. Skeletal histogenesis involves overt differentiation of cells that secrete the extracellular matrix of cartilage and bone (chondrocytes and osteoblasts, respectively), and follows the mesenchymal condensation of skeletogenic cells. Skeletal morphogenesis directs such differentiation events in space and time. While many studies propose a molecular genetic basis for evolutionary changes to skeletal morphogenesis [1-5], the evolution of skeletal histogenesis among vertebrates is fertile ground for additional molecular analyses [6,7].

**Figure 1:**
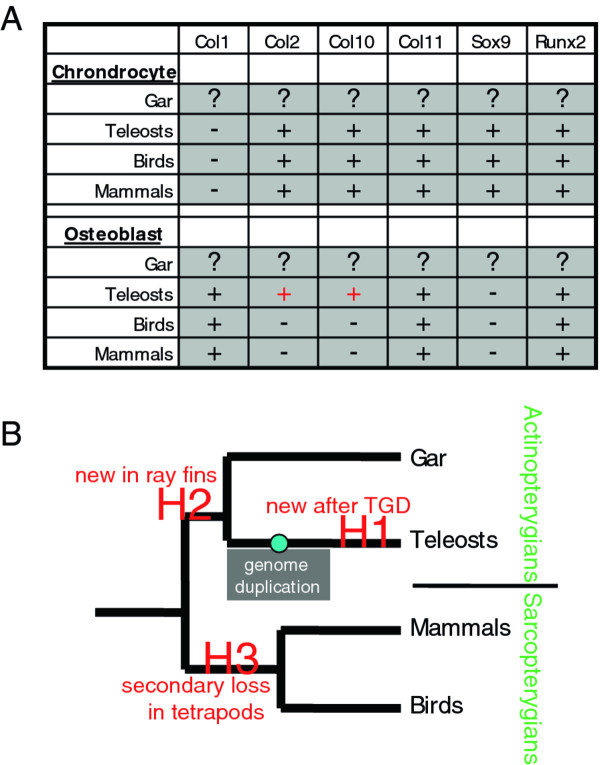
**Comparing molecular fingerprints of skeletal cells suggests evolution of the osteoblast among vertebrate clades**. **A**. Published collagen and transcription factor gene expression in various vertebrate clades reveals variation in the molecular fingerprint of the osteoblast (red text), whereas the chondrocyte shows a conserved molecular fingerprint. References and species cited: Teleosts = *A. semicinctus, B. horae, C. aceratus, C. aeneus, D. rerio, G. aculeatus, G. aymonieri, J. floridae, M. sanctaefilomenae, O. latipes, P. antarcticum, P. pangasius, P. pungitius, P. reticulate, R. trilineata, T. ladigesi *[11-14]; Mammals = *H. sapiens, M. musculus, R. norvegicus *[15-18]; Birds = *A. platyrynchos, C. coturnix japonica, G. gallus *[15,19-22]. Almost every reference cited in this table focused on cranial skeletal tissues, but published work suggests that the summarized molecular fingerprints can be applied to skeletal cells throughout the body [19]. **B**. Three possible evolutionary scenarios explain the expression of Col2 and Col10 in osteoblasts of Teleosts, but not Tetrapods (Mammals + Birds). Hypothesis 1 suggests neofunctionalization (ectopic expression of Col2 and Col10) in osteoblasts appeared after the teleost-specific genome duplication, or TGD. Hypothesis 2 suggests neofunctionalization in osteoblasts appeared in the ancestral Actinopterygian, or ray-finned fish. Hypothesis 3 suggests Col2 and Col10 expression was present in the common ancestor of Actinopterygians and Sarcopterygians (i.e. ancestral Osteichthyan), and was subsequently lost in the Sarcopterygian (lobe-finned fish) lineage. Abbreviations: Col1 = Collagen type 1a2; Col2 = Collagen type 2a1; Col10 = Collagen type 10a1; Col11 = Collagen type 11a2.

Each cell type achieves and performs its function by employing a specific set of genes, recently termed its molecular fingerprint [6,8,9]. Are molecular fingerprints free to evolve in each vertebrate clade in response to selective pressures? Or were molecular fingerprints fixed shortly after tissues first evolved and since then have remained rather constant? For example, skeletons of land animals evolved under greater gravitational stress than skeletons of aquatic animals [10]. As a result, one might hypothesize that the molecular fingerprints of skeletal cells in tetrapods, for instance, would differ from those of teleosts. Alternatively, molecular fingerprints of skeletal cells may have been fixed at the evolutionary origin of the skeleton, and other mechanisms may adapt animals to lineage-specific selective pressures. Comparative studies can reveal the extent to which molecular fingerprints of skeletal cells evolved among vertebrate clades, although the few clades examined thus far are biased towards crown-groups of both ray-finned (actinopterygian) and lobe-finned (sarcopterygian) fish (see Figure 1). Therefore, analyses of more basally-diverging lineages, such as gar or lungfish, will provide evolutionary perspective to these studies.

Comparisons among human, mouse, and chick skeletal tissues suggest that the molecular fingerprints of chondrocytes or osteoblasts do not vary greatly among tetrapods (Figure 1A). Tetrapods exhibit one type of bone tissue, but they have three types of cartilage: elastic cartilage, hyaline cartilage, and fibrocartilage [6,23,24]. Here, we focus on the predominant type of cartilage in vertebrates, hyaline cartilage, which serves as the template for bone during endochondral ossification. Collagens are the most abundant proteinaceous skeletal matrix components, and tetrapod chondrocytes and osteoblasts typically express different fibrillar collagens. In tetrapods, Collagen type 1 alpha 2 (Col1a2) is expressed abundantly in bone and is absent from cartilage, while Collagen type 2 alpha 1 (Col2a1) typifies cartilage and is not expressed in tetrapod bone [23,24]. Hyaline cartilage chondrocytes undergo a maturation process during development, when they express Collagen type 10 alpha 1 (Col10a1), but tetrapod bone does not express Col10a1 [15,25]. Despite these fundamental differences in collagen expression of tetrapod osteoblasts and chondrocytes, their molecular fingerprints also overlap; for example, both cell types express Collagen type 11 alpha 2 (Col11a2; [26]).

Skeletogenic transcription factors control molecular fingerprints of chondrocytes and osteoblasts; Sox9 is required for chondrocyte differentiation, while Runx2 is necessary for osteoblast differentiation [15]. Sox9 and Runx2 dictate skeletal cell differentiation by binding to and promoting the transcription of genes that impart identity to skeletal tissues. For instance, Sox9 directly regulates *Col2a1 *expression, while Runx2 activates *Col1a2 *transcription [27,28]. Much of the overlap in molecular fingerprints of tetrapod chondrocytes and osteoblasts can be attributed to Runx2 activity, which is required for chondrocyte maturation in addition to its role in osteoblast differentiation [29,30]. Perhaps such overlap is not surprising, considering that tetrapod chondrocytes and osteoblasts differentiate from a bipotential progenitor cell, the osteochondroprogenitor, during both embryonic and adult stem cell development [31,32]. Indicative of the delicate balance required for these transcription factors to direct discrete cell lineages, Sox9 can repress Runx2 activity [31,32].

Although hyaline cartilage chondrocytes show conserved molecular fingerprints among vertebrate clades, a few studies in fish suggest that the molecular fingerprint of osteoblasts varies among vertebrates (Figure 1A). In contrast to tetrapod osteoblasts, zebrafish osteoblasts express *col10a1*, and various teleosts show evidence of Col2 in their bone matrix [11,12,33,34]. A lineage-specific genome duplication event, the teleost genome duplication (TGD, or R3), occurred at the base of the teleost radiation, and genome duplications have been thought to facilitate the origin of new gene capabilities [35-40]. For example, Sox9 does not have a direct effect on osteoblast differentiation in tetrapods, but a Sox9 duplicate in teleosts (*sox9b*) has been reported to affect bone development [41]. Therefore, one of at least three evolutionary scenarios might explain differences in the molecular fingerprint of tetrapod and teleost osteoblasts (Figure 1B). Hypothesis 1 proposes that a new function for these collagens in osteoblasts evolved in the teleost lineage, perhaps facilitated by the TGD (i.e., neofunctionalization). Hypothesis 2, like Hypothesis 1, proposes that collagen gene neofunctionalization occurred, but that this event happened in the ancestral actinopterygian osteoblast, and hence ruling out the hypothesis that the TGD facilitated this novel gene function. Hypothesis 3 proposes that the last common ancestor to both tetrapods and teleosts had osteoblast expression patterns found in today's teleosts, but that these patterns were lost secondarily in the tetrapod lineage (Figure 1B). To distinguish among these possibilities, we compared molecular fingerprints of skeletogenic cells in the teleost *Danio rerio *with the spotted gar *Lepisosteus oculatus*, a member of a teleost sister group that diverged before the TGD. Currently, the molecular fingerprints of chondrocytes and osteoblasts are completely unknown for any non-teleost actinopterygian.

Results demonstrated that gar and zebrafish share molecular fingerprints of both chondrocytes and osteoblasts. As an indication of skeletal cell molecular fingerprints, we used *in situ *hybridization on developing gar and zebrafish embryos. Specifically, we analyzed expression of the structural collagen genes *col1a2, col2a1, col10a1*, and *col11a2 *in well-developed cartilage and bone, and also revealed expression of the transcription factor genes *sox9 *and *runx2 *during mesenchymal condensation. We found that, like osteoblasts in the teleost *Danio rerio*, gar osteoblasts expressed *col2a1 *and *col10a1*. Therefore, these data refute by parsimony the role of the TGD in the origin of lineage-specific skeletal molecular fingerprints (Hypothesis 1) and furthermore argue that the expression of "chondrocyte" genes in osteoblasts is a shared feature of actinopterygians. More experiments will be required to distinguish between Hypotheses 2 and 3. In efforts to explain the actinopterygian expression patterns reported here, we found, surprisingly, that the "chondrogenic" transcription factor *sox9 *was expressed in developing gar and zebrafish osteoblasts. We discuss these findings in a phylogenetic context and suggest that the molecular fingerprint of the primitive vertebrate osteoblast was less fixed than previously expected from studies of tetrapods.

## Methods

### Fish

All fish and embryos were maintained with IACUC approval, according to established protocols [42,43]. Wild-type zebrafish were of the AB strain; gar originated from animals collected in Lafourche Parish, Louisiana (courtesy of Drs. A. Ferrara and Q. Fontenot).

### Histology and confocal microscopy

Embryos and larvae were processed for Alcian blue/Alizarin red staining and sectioned for histology as described previously [44]. For zebrafish, we used transgenic lines to help visualize the location and organization of specific populations of cells. *Tg(foxp2.A:EGFP)zc42 *fish produce chondrocyte fluorescence [45], *Tg(sp7:EGFP)b1212 *fish make fluorescent osteoblasts [46], and *Tg(fli1a:EGFP)y1 *fish have fluorescence broadly among neural crest cells of the head [47]. Animals were imaged live under a confocal microscope while stained with the vital dye Alizarin red, as described previously [44].

### Molecular cloning and section in situ hybridization

Whole-mount and section RNA *in situ *hybridization were carried out as described [41,48]. Zebrafish probes used were *runx2a, runx2b, sox9a, sox9b, col1a2, col2a1a, col10a1*, and *col11a2 *[13,49].

## Results

### Early and late stages of ceratohyal and dentary development

**Figure 2:**
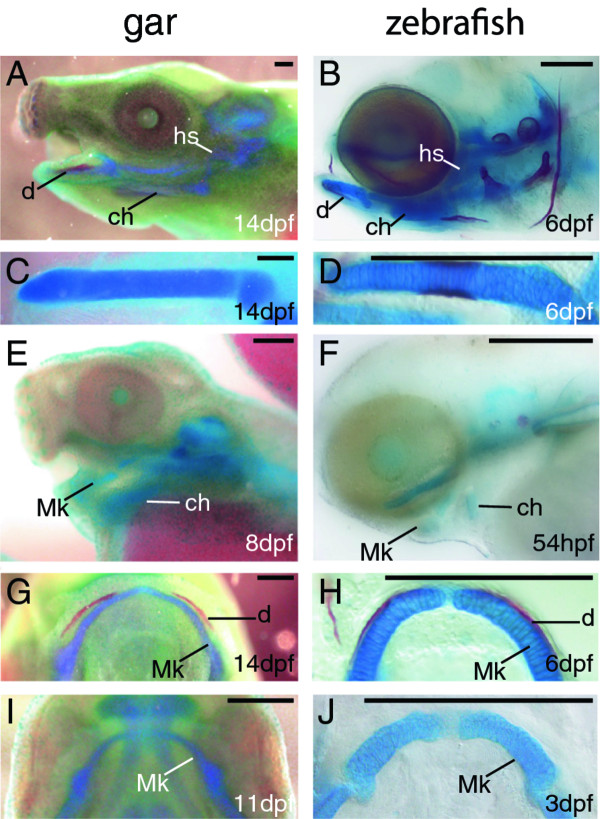
**Temporal series of skeletal preparations reveal early and late stages of cartilage and bone development in gar and zebrafish**. **A-J**, Alcian blue (cartilage)/Alizarin red (mineralized bone) stained fish larvae. Lateral images of larval head skeletons show that many cartilage and a few bone elements were well-formed by 14 dpf in gar (A) and by 6 dpf in zebrafish (B). Dissected and flat-mounted ceratohyals of 14 dpf gar (C) and 6 dpf zebrafish (D) showed strong Alcian blue staining and distinct boundaries of the skeletal element, which are two features of well-developed cartilage. At earlier stages, more faint and diffuse Alcian blue staining was apparent in developing ceratohyal condensations of 8 dpf gar (E) and 54 hpf zebrafish (F). Ventral views show obvious Alizarin red staining of the dentary along anterolateral aspects of Meckel's cartilage in the lower jaws of 14 dpf gar (G) and 6 dpf zebrafish (H). At earlier timepoints, no Alizarin red staining was visible in regions of the dentary in 11 dpf gar (I) and 3 dpf zebrafish (J). Scale bars: A-J = 0.25 mm. Abbreviations: ch = ceratohyal; d = dentary; dpf = days post-fertilization; hpf = hours post-fertilization; hs = hyosymplectic; Mk = Meckel's.

To elucidate molecular fingerprints of chondrocytes and osteoblasts in gar and zebrafish, we focused on two cranial skeletal elements: the ceratohyal, which in mammals forms the anterior horn of the hyoid bone and whose chondrocytes form directly from mesenchyme during endochondral ossification; and the dentary, which in mammals forms the mandible bone and whose osteoblasts differentiate directly from mesenchyme during intramembranous ossification. To define equivalent developmental stages of cranial skeletogenesis between gar and zebrafish larvae, we performed whole-mount staining with Alcian blue and Alizarin red on a variety of stages for both species (Figure 2A, B). Our histological analyses of ceratohyal development demonstrated a well-defined, Alcian blue-stained cartilage rod in gar larvae by 14 days post-fertilization (dpf) and in zebrafish larvae by 6 dpf (Figure 2A-D). In the same region earlier in development, a faint Alcian blue-stained condensation of mesenchyme was apparent in gar (8 dpf) and zebrafish (54 hours post-fertilization; hpf) (Figure 2E, F). Dentary bone formation was evidenced by substantial Alizarin red staining adjacent to the anterolateral aspects of Meckel's cartilage at 14 dpf for gar and at 6 dpf for zebrafish (Figure 2A, B, G, H). No Alizarin red staining was found in the region of the dentary in gar at 11 dpf or in zebrafish at 3 dpf (Figure 2I, J). These results established the location and timing of equivalent stages of cartilage and bone development in gar and zebrafish.

### Chondrocyte molecular fingerprint

**Figure 3:**
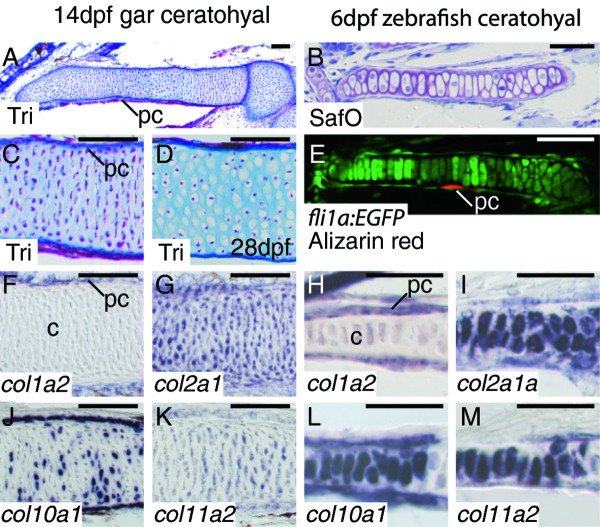
**Collagen expression is equivalent in chondrocytes of gar and zebrafish**. **A, C, D**, Trichrome-stained coronal gar sections. **B**, Safranin O-stained coronal zebrafish section. **E**, confocal slice of ceratohyal in *fli1a:EGFP *zebrafish, which have GFP expression in skeletogenic neural crest cells [47] and Alizarin red staining of calcified bone matrix. **F-M**, *in situ *hybridization on coronal sections. Trichrome staining of the 14 dpf gar ceratohyal (A) shows hundreds of chondrocytes, which had not yet undergone hypertrophy (B), and a thin layer of Aniline blue-stained bone matrix in the perichondrium. Mid-diaphyseal gar chondrocytes had undergone hypertrophy by 28 dpf (C). Safranin O staining identifies cartilage matrix of the 6 dpf zebrafish ceratohyal (B), while confocal imaging of *fli1a:EGFP *transgenic zebrafish ceratohyal (E) illustrates chondrocyte morphology and bone matrix deposition (Alizarin red) in the perichondrium. At these timepoints, chondrocytes in both gar and zebrafish failed to express *col1a2 *(F, H), whereas transcripts for *col2a1 *(G, I), *col10a1 *(J, L), and *col11a2 *(K, M) were detected in mid-diaphyseal mature chondrocytes. All of these collagen genes were expressed in developing perichondrium of both gar and zebrafish ceratohyal. Scale bars: A-M = 50 μm. Abbreviations: c = cartilage; dpf = days post-fertilization; pc = perichondrium; SafO = Safranin O; Tri = Trichrome.

To analyze cellular and molecular features of developing chondrocytes in gar and zebrafish, we examined histologically stained sections, imaged transgenic zebrafish by confocal microscopy, and studied gene expression patterns in tissue sections. At 14 dpf, the gar ceratohyal contained hundreds of chondrocytes, none of which showed evidence of hypertrophy (Figure 3A, C). By 28 dpf, however, the gar ceratohyal showed clearly hypertrophic chondrocytes in the mid-diaphyseal region (Figure 3D). At both 14 dpf and 28 dpf, Aniline blue-stained bone matrix was apparent in an extremely thin layer of the perichondrium. The 6 dpf zebrafish ceratohyal had dozens of chondrocytes, most of which already displayed evidence of hypertrophy (Figure 3B, E; [44]). Alizarin red staining of bone matrix in the perichondrium was also evident. Therefore, well-developed ceratohyals of both the gar and zebrafish displayed equivalent cellular and histological features of chondrocyte (and perichondral bone) development.

We next sought to explore the molecular fingerprints of well-developed chondrocytes in gar and zebrafish. In both gar and zebrafish, *col1a2 *expression was absent or very low in chondrocytes of the well-formed ceratohyal, although it was expressed clearly in cells of the perichondrium of both species (Figure 3F, H). Ceratohyals in both gar and zebrafish had high levels of *col2a1 *transcripts (Figure 3G, I), although more mature, mid-diaphyseal chondrocytes appeared to have down-regulated transcript levels (data not shown), which is consistent with similar findings in tetrapods [50]. In addition, *col2a1 *expression was detected in the perichondrium of the ceratohyal in both gar and zebrafish. Expression of *col10a1 *was high in mature chondrocytes and in perichondral cells of both gar and zebrafish ceratohyals, whereas surrounding, less mature chondrocytes did not express *col10a1 *(Figure 3J, L). Transcripts for *col11a2 *were evident in both chondrocytes and perichondral cells of gar and zebrafish ceratohyals, although the levels of expression in mature chondrocytes appeared to be reduced relative to adjacent chondrocytes (Figure 3K, M, and data not shown), which again is consistent with published reports in tetrapods [51].

**Figure 4:**
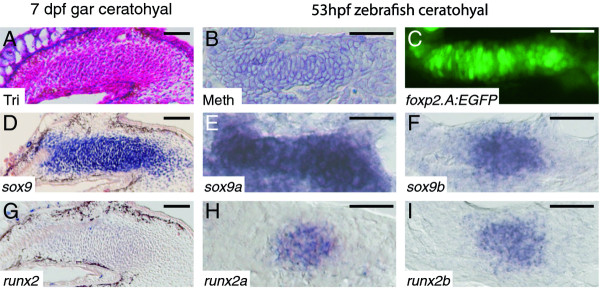
**Chondrogenic condensations of gar and zebrafish display similar transcription factor expression patterns**. **A**, Trichrome-stained coronal gar section. **B**, Methylene blue-stained coronal zebrafish section. **C**, confocal slice of *foxp2.A:EGFP *zebrafish, which express GFP in developing chondrocytes [45]. **D-I**, *in situ *hybridization on coronal sections. Trichrome staining of the 7 dpf gar ceratohyal (A) and Methylene blue staining of the 53 hpf zebrafish ceratohyal (B) show mesenchymal condensation. Confocal imaging of *foxp2.A:EGFP *transgenic zebrafish ceratohyal (C) illustrates condensation of chondrogenic cells at 53 hpf. Similar to expression of *sox9 *in the gar ceratohyal (D), zebrafish ceratohyal expressed both *sox9a *(E) and *sox9b *(F) co-orthologs. While transcripts for *runx2 *were slightly above background in the 7 dpf gar ceratohyal (G), both *runx2a *(H) and *runx2b *(I) co-orthologs were expressed highly in 53 hpf zebrafish ceratohyal. Scale bars: A-I = 30 μm. Abbreviations: dpf = days post-fertilization; hpf = hours post-fertilization; Meth = Methylene blue; Tri = Trichrome.

To help explain the collagen gene expression patterns observed in cells of the well-developed ceratohyal cartilage, we analyzed expression of genes encoding Sox9 and Runx2, two transcription factors known to regulate these collagen genes in tetrapods [15,27,28,52,53]. Because specification of cell types occurs prior to their overt differentiation and transcription factor expression at this timepoint predicts skeletal cell fates [19,31], we focused on the mesenchymal condensation phase of cartilage development. Progenitor cells of the gar ceratohyal had undergone mesenchymal condensation by 7 dpf, while chondrogenic condensation of the zebrafish ceratohyal had occurred by 53 hpf (Figure 4A-C). In gar, *sox9 *transcripts were abundant in chondrogenic cells, whereas levels of *runx2 *expression were only slightly above background (Figure 4D, G). Due to the TGD, zebrafish has two co-orthologs of both *sox9 *and *runx2 *[37,41,54]. Expression patterns of *sox9 *and *runx2 *genes in cells of the zebrafish ceratohyal condensation at 53 hpf were similar to those seen in gar, although levels of *runx2 *gene expression were much higher (Figure 4E, F, H, I). In total, these data demonstrate that gar and zebrafish share molecular fingerprints of developing chondrocytes (Table 1).

**Table 1:** Phylogenetic comparison of chondrocyte molecular fingerprint

Chondrocyte	gar	zebrafish	chick	mouse
*collagen type 1a2*	-	-	-	-

*collagen type 2a1*	+	+	+	+

*collagen type 10a1*	+	+	+	+

*collagen type 11a2*	+	+	+	+

*sox9*	+	+	+	+

*runx2*	+/-	+	+	+

The detection of particular gene expression by *in situ *hybridization is indicated as follows: '+' = high detectable expression; '-' = undetectable expression; '+/-' = low detectable expression. Gar and zebrafish data are presented here, and chick and mouse data come from references cited in Figure 1

### Osteoblast molecular fingerprint

**Figure 5:**
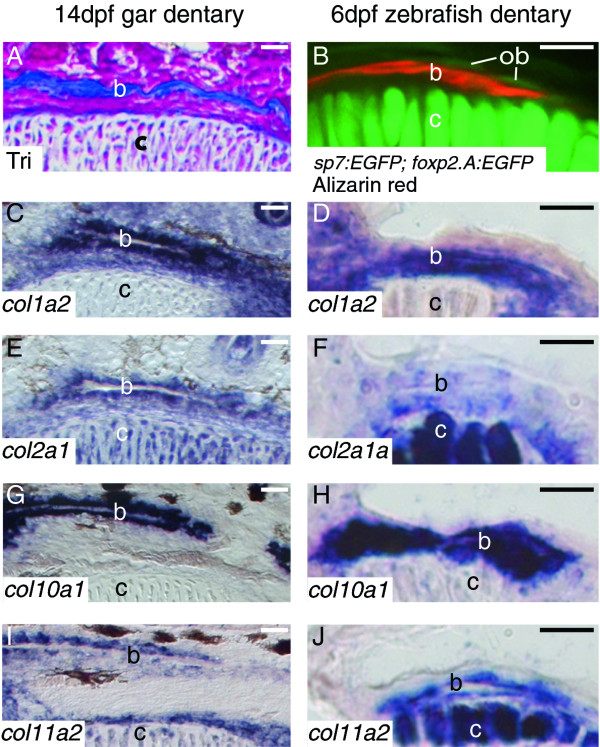
**Collagen expression in osteoblasts of gar and zebrafish is equivalent**. **A**, Trichrome-stained coronal gar section. **B**, confocal slice of *sp7:EGFP; foxp2.A:EGFP *zebrafish, which have GFP expression in both developing osteoblasts and developing chondrocytes, respectively [45,46] and also are stained with Alizarin red to visualize mineralized bone matrix. **C-J**, *in situ *hybridization on coronal sections. Aniline blue staining in the 14 dpf gar (A) and Alizarin red staining in the 6 dpf zebrafish (B) reveals bone matrix of the dentary. Osteoblasts of the zebrafish dentary are labeled with the *sp7:EGFP *transgene. Osteoblasts of both the gar and zebrafish dentaries expressed *col1a2 *(C, D), *col2a1 *(E, F), *col10a1 *(G, H), and *col11a2 *(I, J), although expression of *col2a1a *in zebrafish osteoblasts was relatively lower than seen in gar osteoblasts. Scale bars: A-J = 15 μm. Abbreviations: b = bone; c = cartilage; dpf = days post-fertilization; ob = osteoblast; Tri = Trichrome.

To analyze the cellular and molecular features of developing osteoblasts in gar and zebrafish, we performed histological stains, confocal imaging of transgenic zebrafish, and *in situ *hybridization on tissue sections. The dentaries of 14 dpf gar and 6 dpf zebrafish showed abundant bone matrix adjacent to Meckel's cartilage (Figure 5A, B). In both gar and zebrafish, *col1a2 *expression was high in osteoblasts of the well-developed dentary (Figure 5C, D). Transcripts for *col2a1 *were apparent in both gar and zebrafish dentaries, although levels detected in the zebrafish were relatively lower (Figure 5E, F). Osteoblasts of both gar and zebrafish dentaries demonstrated abundant *col10a1 *expression, as well as high levels of *col11a2 *transcripts (Figure 5G-J).

**Figure 6:**
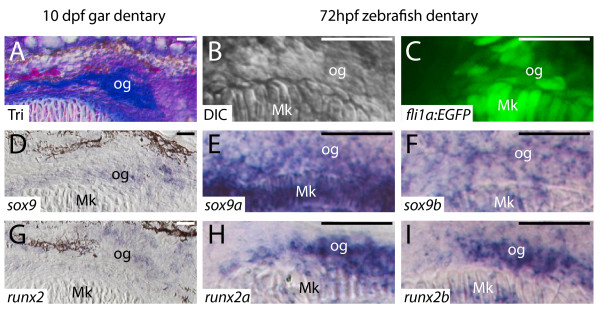
**Transcription factor expression in osteogenic condensations of gar and zebrafish is equivalent**. **A**, Trichrome-stained coronal gar section. **B**, Differential interference contrast image of zebrafish. **C**, confocal slice of *fli1a:EGFP *zebrafish. **D-I**, *in situ *hybridization on coronal sections. Osteogenic cells of the 10 dpf gar dentary have begun to secrete Aniline blue-stained bone matrix, but do not stain with Alizarin red, as this new matrix is uncalcified (data not shown). Osteogenic cells of the 72 hpf zebrafish dentary are located in an equivalent position. Similar to expression of *sox9 *in the gar dentary (D), the zebrafish dentary expressed both *sox9a *(E) and *sox9b *(F) co-orthologs. Osteogenic cells of the gar dentary expressed transcripts for *runx2 *(G), and osteogenic cells of the zebrafish dentary expressed both *runx2a *(H) and *runx2b *(I) co-orthologs. Scale bars: A-I = 20 μm. Abbreviations: DIC = differential interference contrast; dpf = days post-fertilization; hpf = hours post-fertilization; Mk = Meckel's; og = osteogenic cells; Tri = Trichrome.

To help understand the expression patterns of collagen genes observed in osteoblasts of the well-developed dentary bone, we again analyzed *sox9 *and *runx2 *expression during mesenchymal condensation. At 10 dpf, osteogenic cells of the gar dentary had undergone mesenchymal condensation, and were beginning to secrete Aniline blue-stained bone matrix (Figure 6A). Osteogenic cells of the zebrafish dentary condensation were visible at 72 hpf (Figure 6B, C). Both *sox9 *and *runx2 *transcripts were apparent in osteogenic cells of the gar dentary (Figure 6D, G). The expression of *sox9 *in osteogenic cells of the gar dentary was dynamic during development. While *runx2 *transcript levels were high in presumptive pre-osteoblasts lateral to Meckel's cartilage at 7 dpf, *sox9 *expression was not detected in these cells (Additional file 1: Figure S1). These data argue that *sox9 *expression increased in osteoblasts as they began to differentiate. Similar to the single *sox9 *gene in gar, both *sox9a *and *sox9b *were expressed in osteogenic cells of the developing zebrafish dentary at 72 hpf (Figure 6D-F). Also overlapping with the single *runx2 *expression in the gar dentary, *runx2a *and *runx2b *transcripts were abundant in the zebrafish dentary at 72 hpf (Figure 6G-I). In summary, these data demonstrate that gar and zebrafish share molecular fingerprints of developing osteoblasts (Table 2).

**Table 2:** Phylogenetic comparison of osteoblast molecular fingerprint

Osteoblast	gar	zebrafish	chick	mouse
*collagen type 1a2*	+	+	+	+

*collagen type 2a1*	+	+/-	-	-

*collagen type 10a1*	+	+	-	-

*collagen type 11a2*	+	+	+	+

*sox9*	+	+	-	-

*runx2*	+	+	+	+

The detection of particular gene expression by *in situ *hybridization is indicated as follows: '+' = high detectable expression; '-' = undetectable expression; '+/-' = low detectable expression. Gar and zebrafish data are presented here, and chick and mouse data come from references cited in Figure 1

## Discussion

Due to their preservation in the fossil record, cartilage and bone serve as invaluable traits in understanding vertebrate evolution. Evolutionary inferences, however, often assume that the histogenesis of skeletal tissues themselves remains constant among vertebrate lineages. To be fair, cells that produce cartilage and bone (i.e., chondrocytes and osteoblasts, respectively) may have been free to evolve since their appearance roughly 500 million years ago [55,56]. Here, we ask explicitly: To what extent do vertebrate clades share expression of the sets of genes that characterize skeletogenic cell types (i.e., molecular fingerprints; [6,8,9])?

A molecular fingerprint that is shared among vertebrate clades would suggest evolutionary constraints on that skeletal cell type (i.e., skeletal cell types are not free to vary). For instance, cells in cranial and appendicular skeletal tissues have different embryologic origins, and so developmental constraints may limit the molecular fingerprint of a skeletal cell that appears in both regions. While future experiments can test this hypothesis more extensively, skeletogenic cells in different embryonic regions (i.e., cranial vs. appendicular) of a given individual have been shown to exhibit a conserved molecular fingerprint [19]. Another interesting potential embryonic constraint is the fact that osteoblasts have two evolutionary and developmental origins within vertebrates. During vertebrate phylogeny, bone originated in the dermis (i.e., exoskeleton), and then later appeared in the perichondrium surrounding cartilage templates (i.e., endoskeleton) [57]. While not a focus of this study, we did not find differences between molecular fingerprints of osteoblasts from the exoskeleton (e.g., those in the dentary) and endoskeleton (e.g., those surrounding the ceratohyal). Therefore, our results do not support the notion that the exoskeleton and endoskeleton have separate embryonic constraints on the molecular fingerprints of osteoblasts, but testing this hypothesis could be a fruitful avenue of future research.

A molecular fingerprint that varies among clades suggests relaxed constraints on the evolution of that cell type. One might expect variation in molecular fingerprints of skeletogenic cells among various vertebrate lineages, especially given the different selective pressures to which each vertebrate clade has been exposed. For example, the skeletons of land animals withstand a stronger effective gravitational force than do the skeletons of water-borne animals [10]. Some aquatic lineages, including sharks and other "cartilaginous" fish, and some Antarctic fish, have even lost the majority of their bony skeleton at some point during phylogeny [13,58]. Are signatures of the embryonic response to these varied selective pressures seen in the molecular fingerprints of skeletogenic cells across vertebrates?

Spurred by the reported and unexpected expression of *col10a1 *and Col2, two markers of tetrapod chondrocytes, in osteoblasts of teleosts (Figure 1, Tables 1, 2; [11,12]), we pursued the hypothesis that molecular fingerprints of skeletogenic cells vary among vertebrate clades. Experiments revealed collagen and transcription factor gene expression in skeletal cells of hyaline cartilage and bone in the zebrafish--a teleost--and gar, which diverged in the actinopterygian lineage prior to the teleost-specific genome duplication (TGD; Figure 1; [43]). Specifically, our data distinguish among competing hypotheses to explain why osteoblasts of teleosts express *col10a1 *and Col2, which are not expressed in osteoblasts of tetrapods. Osteoblast expression of these collagens either represents a neofunctionalization event that was specific either to the teleost lineage subsequent to the TGD (Hypothesis 1) or to the actinopterygian lineage (Hypothesis 2), or they were expressed in osteoblasts of the common ancestor of tetrapods and teleosts and subsequently lost in tetrapods (Hypothesis 3, Figure 1). Admittedly, evaluation of molecular fingerprints based upon expression of a few genes is a limited approach, but our findings on gene expression in chondrocytes and osteoblasts of the gar and zebrafish suggest evolutionary trends that could be embellished by massively parallel transcriptomics (e.g., RNA-seq; [59]).

We demonstrate that gar and zebrafish share molecular fingerprints of both chondrocytes and osteoblasts (Tables 1, 2). Despite evidence that genome duplication can facilitate the origin of new gene functions [35,36,39,40], our data reject the proposed teleost neofunctionalization hypothesis for osteoblast evolution (Hypothesis 1, Figure 1). Because both gar and zebrafish express *col2a1 *and *col10a1 *in their osteoblasts, the most parsimonious explanation is that these markers were present in the molecular fingerprint of the ancestral actinopterygian osteoblast. Therefore, parsimony favors Hypothesis 2, although our results do not reject Hypothesis 3, and more experiments are required to distinguish between these two possibilities. The notion that osteoblasts achieved collagen neofunctionalization somewhere in the actinopterygian lineage (Hypothesis 2, Figure 1) could be tested further by revealing the molecular fingerprint of osteoblasts in bichir, an actinopterygian diverging more basally than the gar lineage [60]. Similar studies of the lungfish or coelocanth, basally-diverging sarcopterygians, would test the possibility that *col2a1 *and *col10a1 *expression was present in osteoblasts of the ancestral bony fish and subsequently was lost somewhere in the sarcopterygian lineage leading to tetrapods (Hypothesis 3, Figure 1).

Our studies of skeletogenic transcription factors suggest a functional framework to explain why *col2a1 *and *col10a1 *are expressed in osteoblasts of actinopterygians, but not in osteoblasts of sarcopterygians (Table 2). In addition to *runx2 *expression, gar and zebrafish osteoblasts express *sox9 *during mesenchymal condensation of dermal bones. Developing osteoblasts of tetrapods typically express *Runx2 *but not *Sox9 *during mesenchymal condensation of dermal bones [19,31,61]. We propose that the expression of *sox9 *in gar and zebrafish osteoblasts may explain the presence of *col2a1 *and *col10a1 *transcripts, given two assumptions. First, actinopterygian osteoblasts would have to translate the *sox9 *transcript we observed into Sox9 protein. Second, Sox9-responsive cis-acting regulatory elements that drive *Col2a1 *expression in tetrapods [27] would have to operate similarly in actinopterygian lineages. In support of this latter notion, *col2a1 *gene expression is extinguished in *sox9 *mutant zebrafish [41]. Currently, Sox9 has not been shown to bind to the *Col10a1 *promoter, but mis-expression of Sox9 in developing avian osteoblasts causes ectopic *Col10a1 *expression, and loss of Sox9 can abrogate *Col10a1 *expression in mouse [31,62], showing that *Col10a1 *is downstream of Sox9 control. Deciphering the molecular mechanism by which *sox9 *expression in developing osteoblasts can vary among vertebrate clades will shed light on the evolution of cell type-specific molecular fingerprints.

If the primitive condition for osteoblasts in the common ancestor of actinopterygians and sarcopterygians included expression of *sox9, col2a1*, and *col10a1*, then tetrapod osteoblasts would have lost expression of these genes secondarily, as outlined in Hypothesis 3. This possibility would give an entirely fresh phylogenetic context to reports of a transient chondrogenic phase during tetrapod dermal bone development [61,63]. Interestingly, sub-populations of chondrocytes in the zebrafish may lose *sox9 *and *col2a1 *expression as they transition to osteocytes in response to Hh signaling [64], so a developmental precedence may exist for the transitions in molecular fingerprints that Hypothesis 3 proposes during evolution. More broadly, we reveal fundamental differences between the molecular fingerprints of osteoblasts in actinopterygian and sarcopterygian clades, a finding consistent with the hypothesis that the primitive osteoblast-like cell was under reduced constraint (i.e., free to vary) during early vertebrate phylogeny.

Comparison of our data with published data for tetrapods further argues that, while the osteoblast has evolved differently between actinopterygian and sarcopterygian lineages, the molecular fingerprint of the chondrocyte appears to be conserved among vertebrates (Tables 1, 2). Although sampling of vertebrate lineages in this manner is as yet too restricted to be confident of making generalizations, limited studies on chondrocytes of hyaline cartilage in amphibians and reptiles do support this conclusion [65-67].

What mechanisms allow the osteoblast to vary among extant vertebrates, then, but constrain the chondrocyte? We argue that cell types that appear earlier in phylogeny and ontogeny are less free to vary during subsequent evolution. Cartilage appeared in the fossil record in the primitive chordate *Haikouella *530 million years ago, and hyaline cartilage is a shared trait among chordates, hemichordates, and even some disparate protostome taxa [6,56,68]. Apart from two clades of diverged agnathans (i.e., hagfish and lamprey), all vertebrate lineages develop bone, which appeared in fossilized conodonts from 515 million years ago [55,69]. Therefore, cartilage appeared before bone during phylogeny. In addition, cartilage appears before bone during ontogeny. Taken together, we suggest that because the chondrocyte appears before the osteoblast during both phylogeny and ontogeny, the molecular fingerprint of the chondrocyte is more constrained than that of the osteoblast. As such, our interpretation is consistent with the notion of phyletic constraint [70] and may provide a novel system by which to analyze molecular details of a developmental constraint.

## Conclusions

While the molecular genetic basis for evolutionary changes to skeletal morphology has received much attention, similar studies on the evolution of skeletal cell types is limited. The set of genes, or molecular fingerprint, expressed by a cartilage- or bone-forming cell (chondrocyte or osteoblast, respectively) has been determined largely from human, mouse, and chick, thus providing an extremely limited sampling among vertebrate clades. A couple of studies demonstrated that teleost osteoblasts express collagens that normally are expressed only in chondrocytes of tetrapods, allowing us to generate specific hypotheses on the evolution of the osteoblast among vertebrates (Figure 1). Here, we test the hypothesis that the molecular fingerprint of the osteoblast underwent neofunctionalization in the teleost lineage specifically, perhaps as a result of the teleost-specific genome duplication (TGD). We compare expression of collagen and transcription factor genes during embryonic development of cartilage and bone in the teleost zebrafish *Danio rerio *and the spotted gar *Lepisosteus oculatus*, which diverged in the actinopterygian lineage prior to TGD. We find equivalent expression patterns of these genes in chondrocytes and osteoblasts of zebrafish and gar, thus refuting by parsimony the hypothesis. In addition, we show expression of the "chondrocyte" transcription factor *sox9 *in developing osteoblasts of zebrafish and gar, providing a molecular explanation for the expression of "chondrocyte" genes in fish osteoblasts. Finally, we argue from comparing our results to those of tetrapods that the molecular fingerprint of the osteoblast was not fixed during early vertebrate evolution, which supports previous work on bone and dentine tissues in the fossil record [57,71], whereas the molecular fingerprint of the hyaline chondrocyte is constrained among vertebrate clades.

## Authors' contributions

BFE, AA, and JHP contributed to the conception of this study. BFE, AA, YLY, and JHP designed the experiments. BFE stained bone and cartilage of staged specimens. AA cloned gar genes. YLY performed *in situ *hybridizations. BFE generated all images and wrote the manuscript. All authors read, revised, and approved the final manuscript.

## Supplementary Material

Additional file 1 **Figure S1**. Transcription factor expression in pre-osteoblasts of gar. **A**, Trichrome-stained coronal section. **B, C**, *in situ *hybridization on coronal sections. Trichrome staining of 7 dpf gar (A) shows mesenchymal cells lateral to the condensation of Meckel's cartilage. These pre-osteoblasts do not express *sox9 *(B), but express high levels of *runx2 *(C). Scale bars: A-C = 50 μm. Abbreviations: dpf = days post-fertilization; Mk = Meckel's; po = pre-osteoblasts; Tri = Trichrome. Click here for file
