# Supplementary figures and images for: Evolution of the osteoblast: skeletogenesis in gar and zebrafish
Source: BMC Evol Biol. 2012 Mar 5;12:27. doi: 10.1186/1471-2148-12-27 (PMC3314580; doi:10.1186/1471-2148-12-27)

# 7dpf gar dentary

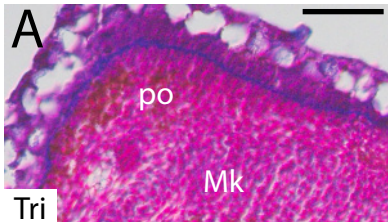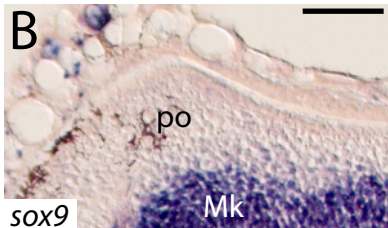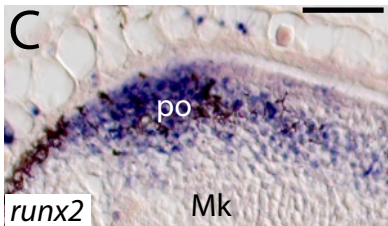

Supplement: Additional file 1 — Figure S1. Transcription factor expression in pre-osteoblasts of gar. A, Trichrome-stained coronal section. B, C, in situ hybridization on coronal sections. Trichrome staining of 7 dpf gar (A) shows mesenchymal cells lateral to the condensation of Meckel's cartilage. These pre-osteoblasts do not express sox9 (B), but express high levels of runx2 (C). Scale bars: A-C = 50 μm. Abbreviations: dpf = days post-fertilization; Mk = Meckel's; po = pre-osteoblasts; Tri = Trichrome. [file 1471-2148-12-27-S1.PDF]
